# Clinical utility of array comparative genomic hybridisation in prenatal setting

**DOI:** 10.1186/s12881-016-0345-8

**Published:** 2016-11-15

**Authors:** Luca Lovrecic, Ziga Iztok Remec, Marija Volk, Gorazd Rudolf, Karin Writzl, Borut Peterlin

**Affiliations:** Clinical Institute of Medical Genetics, University Medical Centre Ljubljana, Slajmerjeva 3, SI-1000 Ljubljana, Slovenia

## Abstract

**Background:**

The objective of reported study was to evaluate the clinical utility of prenatal microarray testing for submicroscopic genomic imbalances in routine prenatal settings and to stratify the findings according to the type of fetal ultrasound anomaly.

**Methods:**

From July 2012 to October 2015 chromosomal microarray testing was performed in 218 fetuses with varying indications for invasive prenatal diagnosis: abnormal karyotype, ultrasound anomalies, pathogenic variant in previous pregnancy or carriership in a parent.

**Results:**

The detection rate in the group of fetuses with ultrasound anomalies was 10,0% for pathogenic copy number variants (CNVs), five of them being larger than 8 Mb and expected to be seen on prenatal karyotype. If only those pathogenic CNVs below the classical karyotype resolution are considered, chromosomal microarray testing provided an additional 7,7% diagnostic yield in here reported series. When stratified according to the ultrasound anomalies, the highest percentage of pathogenic CNVs were detected in the group of fetuses with multiple congenital anomalies (16,7%) and lowest in the group of isolated in utero growth restriction (6,3%). In the group of cases with isolated increased nuchal translucency we identified a small interstitial deletion of 16p24.1 involving *FOXF1* gene. Prenatal aCGH also provided important insights into cases with seemingly balanced chromosomal rearrangements found on prenatal karyotype, where additional pathogenic CNV were discovered.

**Conclusion:**

Prenatal chromosomal microarray testing significantly increases the diagnostic yield when compared with conventional karyotyping. The highest added value is shown in prenatal diagnostics in fetuses with abnormal ultrasound results. Variants of unknown significance and risk factor CNVs present important challenges and should be discussed with parents in advance, therefore pretest counseling prior to prenatal testing is very important.

## Background

Genetic testing in the prenatal period has significant implications for fetuses with ultrasound anomalies. When a structural anomaly is discovered in an unborn fetus, it is important to evaluate if it has a genetic origin and whether potentially other clinically important features might be expected, or if it is an isolated finding with good clinical prognosis after surgical intervention.

Ongoing technological developments and knowledge advancements in the last two decades have expanded the spectrum of possible prenatally detected genomic aberrations. With this progress we are able to detect and clinically interpret smaller and smaller genomic imbalances and comparative genomic hybridisation using microarray technology (arrayCGH, aCGH, chromosomal microarrays, molecular karyotyping) has succesfully replaced classical karyotyping in postnatal and prenatal setting. It is a first tier test in patients with developmental delay, intellectual disability, autism spectrum disorders and/or multiple congenital anomalies, with significant increment in the diagnostic utility [1, 2]. In recent years it is becoming widely applied in the prenatal setting, where it is recommended for routine prenatal diagnostic testing in the fetuses with ultrasound anomalies [3, 4]. A large prospective and retrospective studies have shown a 5–10% increase in the detection of clinically relevant copy number variation in the fetuses with ultrasound anomalies, as compared to conventional karyotyping [5–7]. Furthermore, recently 17 studies were evaluated by Grande et al. [8] to investigate the impact of aCGH testing in the group of fetuses with isolated increased nuchal translucency NT > 3,5 mm. Their review suggested 5% incremental yield of the aCGH analysis over classical karyotyping. A small number of studies have assessed the clinical utility of chromosomal microarrays in all pregnancies which underwent the invasive prenatal procedures and reported a copy number abnormality in 1.7% of fetuses with a normal ultrasound scan where the indication for invasive testing was an advanced maternal age or a positive aneuploidy screening test [7]. Based on that, the authors suggest that the aCGH might be used in all prenatal testing, regardless of indication. Indeed, some countries have implemented just such a consensus approach and established an Ad Hoc Committee to assist with ambiguous results [9].

It is widely accepted that chromosomal microarrays are the first tier test in prenatal settings in fetuses with ultrasound anomalies or/and increased NT > 3,5 mm. At the same time, the inevitable challenge present the potential to detect variants of unknown significance (VOUS), therefore performing the test in all prenatal cases is currently not a general recommendation. The VOUS frequency in all prenatal samples is estimated to be 0.3–1%, depending on resolution and type of the platform used [10, 11].

In Slovenia, the prenatal aCGH is currently performed in the high-risk pregnancy cohort, where the fetal ultrasound examination is abnormal, increased NT >3,5 mm is seen, or de novo balanced translocation detected or where one parent is a carrier of a pathogenic CNV. We report on our diagnostic, counseling and pregnancy outcome experience in the period from July 2012 to October 2015 in 218 prenatal cases.

## Methods

### The samples and the DNA isolation

The prenatal samples of amniotic fluid, chorionic villi, fetal blood and fetal tissue were received in the period from July 2012 through October 2015 mainly from the in-house ultrasound unit of The Division of Gynaecology and Obstetrics and through our Genetic Counseling outpatient clinic, in total 218 cases. Patients/pregnant couples received the pre-test genetic counseling, including discussion about potential finding of VOUS. An informed consent was obtained. All pathogenic CNVs and VOUS were discussed with patients during post-test genetic counseling. In 18 cases there was a known abnormal fetal karyotype (balanced/unbalanced translocation/rearrangement or marker chromosome) or family history of a chromosome rearrangement in a parent or previous pregnancy. The remaining 200 samples were received in line with the other indications and were further categorized into different groups, based on the type and combination of ultrasound anomaly. The DNA was extracted from direct or cultured chorionic villi, direct amniotic fluid, cultured amniocytes, fetal blood or fetal tissue according to the manufacturer’s protocol using Qiagen Mini kit (Qiagen, Valencia, CA). Quality and concentration parameters of the DNA were measured with NanoDrop 2000c spectrophotometer (Thermo Fisher Scientific Inc.) and Qubit 2.0 fluorometer (Life Technologies Inc.).

### Microarrays

Following sample extraction, DNA was processed according to Agilent protocol (Version 7.3 March 2014) using commercially available male and female genomic DNA (Agilent Technologies, Human Reference DNA, Male and Female) or in-house DNA reference mix as a reference DNA. Agilent SurePrint G3 Unrestricted CGH ISCA v2, 8x60K microarrays were used which provide a practical average resolution of 100 kb. Array images were acquired using Agilent laser scanner G2565CA, image files were quantified using Agilent Feature extraction software for Cytogenomics 3.0 and analysed with Agilent Cytogenomics 3.0 software (Agilent Technologies).

### Classification of results

Called CNVs were aligned with known aberrations in publically available databases - ClinGen (http://dbsearch.clinicalgenome.org/search/), DECIPHER (Database of Chromosomal Imbalance and Phenotype in Humans using Ensembl Resources https://decipher.sanger.ac.uk/), ClinVar (http://www.ncbi.nlm.nih.gov/clinvar/), Database of Genomic Variants - DGV (http://dgv.tcag.ca/dgv/app/home), as well as with the in-house database of detected variants and their clinical significance, ascertained by the trained analysts. All called CNVs were classified in the three groups, benign, VOUS and pathogenic, according to ACMG Standards and Guidelines [12]. In addition, some of the discovered and reported CNVs were not related to the phenotype and were therefore classified as secondary findings. We prefer this terminology over "incidental finding", because in the genome-wide aCGH, such findings may be anticipated and cannot be termed "incidental". The possibility of a secondary finding was discussed with the pregnant couple in the pretest counseling session and identified variants were reported back to the family. The CNVs were classified as benign if they were reported in the above mentioned databases as benign, or present in our in-house database in more than 1% of the cases. The pathogenic CNVs were either known microdeletion/microduplication syndromes or large genome copy number gains and losses, described as pathogenic in the scientific literature. Variants classified as VOUS were either already present in cited databases as VOUS or bigger than 200 kb with OMIM gene content. Both the pathogenic and VOUS CNVs were communicated to the parents. The parental blood samples were collected if VOUS or secondary finding was detected in the fetus.

## Results

### The group of fetuses with abnormal fetal karyotype or known carrier status of one of the parents

Four prenatal samples (4/218, 1,8%) were tested because of known carrier status in the pregnant women and 14 (14/218, 6,4%) samples were cases with known abnormal fetal karyotype.

In the first group there were three mothers with a carrier status, one of them had had two pregnancies in the reported period. She is a carrier of a large Xq21q23 duplication and has a mild intellectual disability with no other health related issues or dysmorphic features. The duplication was confirmed in her male fetus, but not in her female fetus in the subsequent pregnancy. The second female has partial *IL1RAPL1* gene (OMIM*300206) deletion and a son and a brother with the same deletion. Both males had mild intellectual disability and behavioural problems. The same deletion was confirmed in her male fetus. The third female is a TAR (thrombocytopenia absent radius syndrome; OMIM#274000) deletion carrier and her previous pregnancy was terminated due to the confirmed TAR syndrome in the fetus. In her following pregnancy, the fetal sample anaysis identified inherited deletion, but not the hypomorphic nucleotide change on the second allele, present also in the father.

The second group consists of 14 cases in which conventional karyotype analysis showed de novo balanced translocation or unbalanced rearrangement inherited from balanced parent translocation (9 cases), or marker chromosome (5 cases). Details are shown in Table 1. Performed aCGH in a fetus with complex, seemingly balanced translocation involving chromosomes 7, 8 and 12 (Case 1) again proved there were no CNVs at the breakpoints (7q,8q,12q), but identified deletion spanning 3,7 Mb in the short arm of the chromosome 12 (12p12.1). Similarly, chromosomes 1 and 5 were involved in the *de novo* translocation in case 5, but the aCGH revealed 2,5 Mb large deletion at 2q24.3.

**Table 1:** Results of conventional karyotyping and aCGH in prenatal cases with translocations or marker chromosomes

Case	Karyotype	aCGH result
1	46,XY,t(7;8;12)(q34;q21.1;q12)dn,inv(9)(p12q13)pat	12p12.1(21,356,582-25,062,714)x1
2	46,xy,t(4;10)(p16.3;q21.2)dn	Normal profile
3	46,XX,t(3;16)(p?14;p13)dn	Normal profile
4	46,XY,del(4)(p15).ish del(4)(p16.3p16.3)(GS10K2/T7-,LSI WHS-)	4p16.3p15.2(72,447–24,041,772)x1
5	46,XY,t(1;5)(q32;q22)dn	2q24.3(163,875,903–166,239,903)x1
6	46,XY,add(20)(q13.3)	7p22.3p14.1(54,185–38,450,394)x3
7	47,XY,+der(13)dn	13q12.11q12.12(20,412,619–23,874,904)x4
8	46,XY,der(6)t(6;11)(q26;p11.2)pat	6q27(164,600,652–170,921,089)x1, 11p15.5p11.2(210,300–44,934,960)x3
9	46,XX,t(3;11)(q21;q14.2)	Normal profile
10	47,XX,+mar[6]/46,XX[50]^a^	Normal profile
11	47 XY,+mar dn.ish idic(15)(D15Z4++)	Normal profile
12	47,XX,+mar dn.ish der(14/22)(cep14/22+)	22q11.1q11.21(17,397,498–18,628,078)x3-4
13	47,XY,+mar[20]/46,XY[30].ish der(Y)(DXYS129/DXYS153+,SRY+,wcpY+,DYZ1+,wcpY+,DYZ1+,TelXq/Yq+)dn	Yp13.32Yp11.2(10,701–6,592,868)1 ~ 2, Yq11.21q12(14,576,544–59,002,403) × 2 ~ 3, Yq12qter(59,028,692–59,335,913) × 1 ~ 2
14	47,XX,+mar mat.ish der (14/22)(D14Z1/D22Z1)x2,(Acro-P-Arms)x2	Normal profile

^a^results from chorionic villi sample; amniocentesis was performed later and no marker chromosome detected. Healthy female was born

Five cases with marker chromosomes were detected using classical karyotyping, four of them were further characterised by FISH. In the remainig case (Case 10, Table 1), a marker was lost on chorionic villi culture. Amniocentesis was performed and no marker chromosome detected. The pregnancy was continued and healthy female was born.

Additional aCGH testing in the cases 11, 12 and 14 (Table 1) did not confirm any unbalanced genomic rearrangement. Further UPD testing was performed for chromosome 14 or 15, depending on the FISH analysis (UPD15 in the case 11 with marker chromosome derived from chromosome 15; UPD14 in the cases 12 and 14 with positive FISH results for centromere 14 or 22). UPD was excluded in all three cases.

### The group of fetuses with ultrasound anomalies

In all of the following reported cases (*n* = 200, 91,7%) rapid aneuploidy QF-PCR test for detection of numerical aberrations of chromosomes 13,18, 21, X and Y was performed and reported as normal. Overall, the aCGH detection rate for causative pathogenic CNVs was 7,0% (14/200) and for VOUS 2,5% (5/200). Details are shown in Table 2. In addition, secondary findings were identified in 7 cases (7/200, 3,5%). These CNVs were part of known microdeletion/microduplication syndromes or single gene deletions with incomplete penetrance and were discussed case by case, by the professional committee within our department before being reported back to the families.

**Table 2:** The details of clinically significant copy number variations in the fetuses with ultrasound anomalies

Case number	Ultrasound findings	aCGH results	CNV size	CNV classification^a^	Related syndrome/gene or literature and comments
1	Structural heart anomaly, Hydrops	arr[hg19] 1p36.33p36.31(779,727–6,377,318)x1 dn	5,8 Mb	P	1p36 deletion syndrome (OMIM#607872)
2	Bilateral radial aplasia	arr[hg19] 1q21.1q21.2(145,415,190–145,799,602)x1 mat	385 kb	P*	TAR syndrome (OMIM#274000)
3	Bilateral radial aplasia	arr[hg19] 1q21.1q21.2(145,415,190–145,799,602)x1	385 kb	P*	TAR syndrome (OMIM#274000)
4	Oral cleft, Contractures of the large joints	arr[hg19] 1q21.1q21.2(146,507,518–147,379,946)x1 mat, 4q35.2(189,247,673–190,552,305)x1 pat	872 kb 1,3 Mb	SF Likely B	1q21.1 deletion syndrome (OMIM#612474), including *GJA5* gene
5	Spina bifida, hydrocephalus, polydactyly	arr[hg19] 2p25.3p22.1(23,938–41,524,241)x3	41,5 Mb	P	Derived from maternal balanced translocation
6	Cystic hygroma	arr[hg19] 2p16.3(51,109,690–51,251,557) × 1	141,8 kb	SF, P**	*NRXN1* gene (OMIM*600565)
7	IUGR	arr[hg19] 2q13(111,442,130–113,065,779) × 1 pat	1,6 Mb	VOUS	2q13 deletion syndrome [21, 22]
8	Ventriculomegaly, ACC	arr 2q33.3q35(208,814,372–219,814,526)x3 dn	11 Mb	P	*De novo*, many genes
9	Upper limb anomalies	arr[hg19] 5p13.2(36,952,801–37,024,752)x1 dn	72 kb	P	CdL syndrome (OMIM#122470)
10	Multiple congenital anomalies	arr[hg19] 6p25.3p25.1(206,749–5,507,458) × 3 dn	5,3 Mb	P	ORPHA1745
11	Ambiguous genitalia (karyotype 46,XY)	arr[hg19] 9p24.3(220,253–1,999,170)x1 mat	1,7 Mb	P	9p24.3 deletion syndrome, 46,XY sex reversal (OMIM#154230)
12	Multicystic kidney	arr[hg19] 15q11.2(22,765,628–23,217,514) × 1 mat	452 kb	VOUS	15q11.2 risk factor locus, inherited from the mother with mild learning diffuculties
13	Cystic higroma, IUGR	arr[hg19] 15q13.2q13.3(30,653,877–32,861,626) × 3 dn	2,2 Mb	LP	8 OMIM genes, DECIPHER cases
14	Pyelectasis, short femur	arr[hg19] 16p13.3(1,917,269–2,527,114) × 3 pat	610 kb	VOUS	Inherited from healthy father
15	Multiple congenital anomalies	arr[hg19] 16p13.12p11.2(14,145,698–29,331,350) × 3 dn	15,2 Mb	P	*De novo*, many genes
16	IUGR, multicystic kidney	arr[hg19] 16p12.2(21,837,492–22,407,931) × 1 mat	570 kb	SF, P**	16p12.1 deletion syndrome (OMIM#136570)
17	Multiple congenital anomalies	arr[hg19] 16p11.2(29,592,783–30,190,568) × 3 dn	610 kb	SF, P**	16p11.2 duplication syndrome (OMIM#614671)
18	Bilateral pes equinovarus	arr[hg19] 16p11.2(29,673,954–30,190,568) × 1 pat	516 kb	SF, P**	16p11.2 deletion syndrome (OMIM#611913)
19	Vertebral anomalies, scoliosis	arr[hg19] 16p11.2(29,673,954–30,190,568) × 1	516 kb	SF, P**	16p11.2 deletion syndrome (OMIM#611913)
20	Increased NT	arr[hg19] 16q24.1(86,211,031–86,649,743) × 1 dn	439 kb	P	*FOXF1* (OMIM*601089)
21	Occipital meningocele	arr[hg19] 18p11.32p11.21(148,963–14,081,887) × 4 dn	13,9 Mb	P	18p tetrasomy (OMIM#614290)
22	Increased NT	arr[hg19] 20p13(60,747–748,964) × 1, 20q13.13q13.33(47,912,240–62,880,583) × 3	688 kb 15,0 Mb	P	Derived from paternal inversion of chromosme 20
23	Bilateral cleft lip and palate	arr[hg19] 22q11.21(20,659,547–21,440,514)x3 pat	781 kb	SF, P**	Atypical 22q11.2 duplication syndrome (OMIM#608363)
24	Hypoplastic nasal bone, cystic formation in abdomen	arr[hg19] Xp22.31(6,488,721–8,097,511) × 2 mat (male fetus)	1,6 Mb	VOUS	Xp22.31 duplication [23]
25	Complex structural heart anomaly	arr[hg19] Xq13.3(74,463,757–74,651,249) × 3 (female fetus)	188 kb	VOUS	*ZDHHC15*(OMIM*300576)

^**a**^P-pathogenic, VOUS –variant of unknown significance, B - benign, SF - secondary finding; P*hypomorphic nucleotide change on the second allele; P**-microdeletion/microduplication with reduced penetrance; IUGR – in utero growth restriction, ACC – agenesis of corpus callosum

Among 14 clinically significant CNVs, five were larger than 8 Mb and therefore expected to be seen if classical prenatal karyotyping had been perfomed. The remaining 9 clinically relevant pathogenic CNVs below the classical karyotype resolution represent an additional 4,6% diagnostic yield in this reported series. In some cases, follow up studies using FISH and classical karyotype analysis were needed in order to elucidate the mechanism and origin of the identified CNV and to be able to estimate the recurrence risk. For example, in the case 22 (Table 2) conventional karyotyping was performed post aCGH as recombinant chromosme 20 was suspected. This was confirmed and parental studies showed a paternal origin (paternal chromosome 20 inversion). The unbalanced rearrangement in case 5 (Table 2) was also shown to be inherited from the maternal balanced translocation.

In all 5 cases of VOUS these were reported back to the parents, as the parents' blood samples are not collected routinely at the time of the prenatal sampling (amniotic fluid, chorionic villi), but are taken later if required. All couples are informed accordingly during the pretest counseling session.

**Fig. 1:**
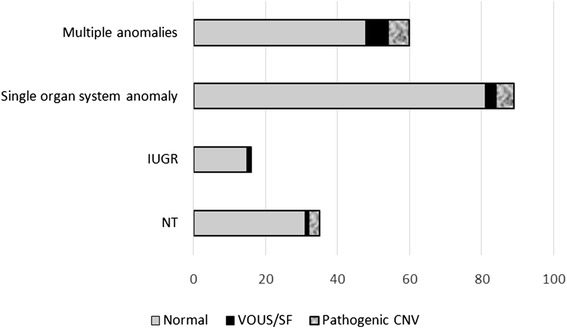
The diagnostic yield of the aCGH in four groups of the prenatal samples, divided according to the categories of ultrasound anomalies. The absolute numbers of cases in each of the four groups are shown on X-axis, according to the aCGH results - normal results (grey), VOUS/secondary findings (black) and causative pathogenic CNVs (marble). Y-axis represents the four groups of prenatal samples according to the ultrasound anomalies/measurements. NT - nuchal translucency, IUGR - intrauterine growth restriction

The two hundred cases of fetuses with ultrasound anomalies were further categorized into the four following subgroups: isolated increased nuchal translucency (NT > 3.5 mm; 35 cases), intrauterine growth restriction (IUGR; 16 cases), single organ system anomaly (89 cases), multiple congenital anomalies (structural anomaly in two or more organ systems; 60 cases). As expected, the highest percentage of pathogenic CNVs was confirmed in the group of fetuses with multiple congenital anomalies (10,0%; 6/60) and the lowest in the group of fetuses with isolated IUGR (0/16), but these numbers are too low to be statistically significant. Further details are presented in the Fig. 1 and Table 2.

**Fig. 2:**
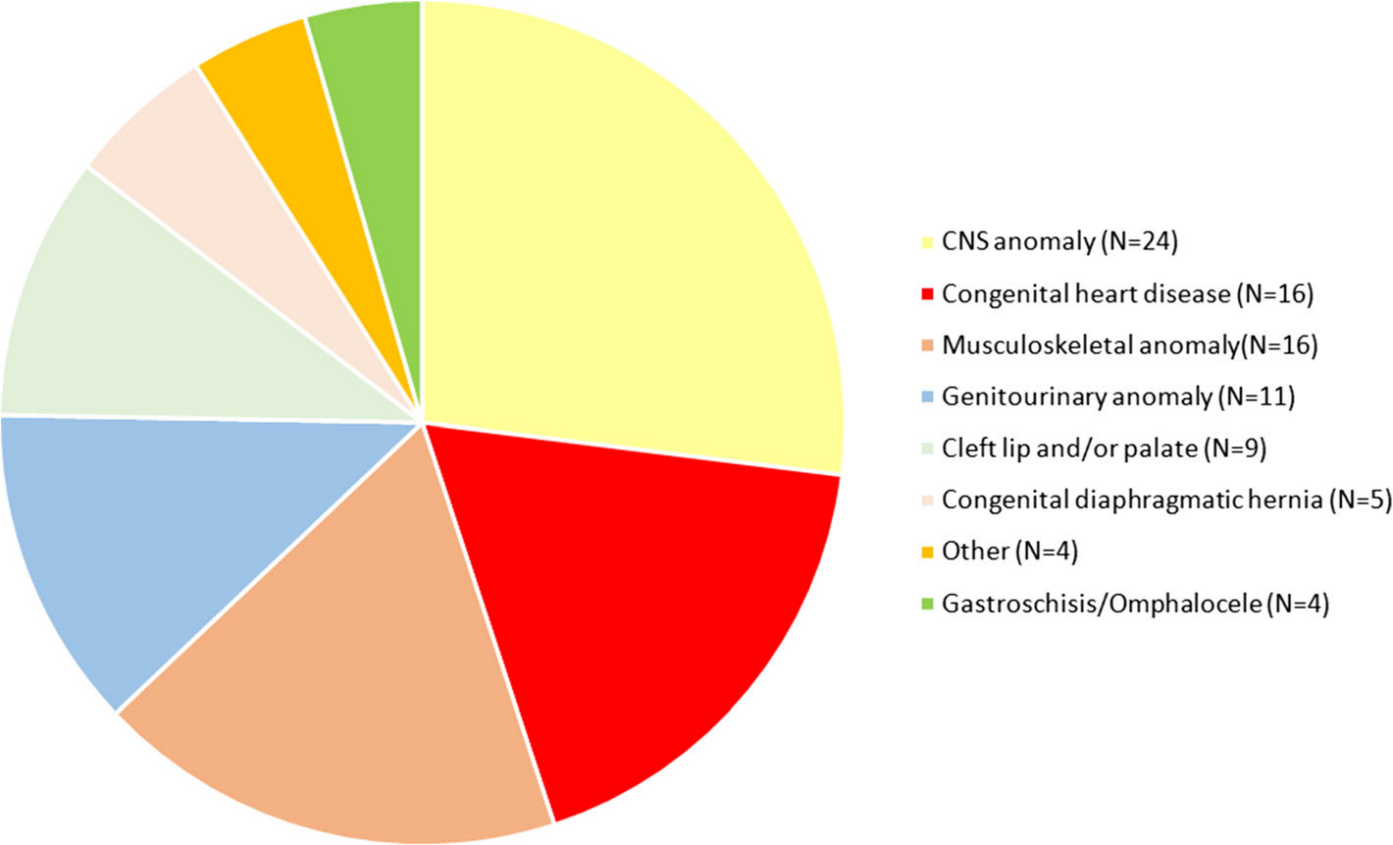
The representation of single system anomalies in the group of the prenatal samples

The heterogeneous group of 89 fetal cases with single organ system anomaly was subcategorized into 7 groups, in order to define common anomalies, as shown in the Fig. 2. Most of the fetuses (63%) had an isolated anomaly of the central nervous system or the heart or the musculoskeletal system. The diagnostic yield of the clinically relevant pathogenic CNVs in the joint group of 89 fetuses was 5,6% (5/89), with the additional 2 cases where VOUS was identified. Only one CNV was big enough to be likely visible using the conventional karyotype analysis.

## Discussion

Diagnostic aCGH has a well established role in the prenatal genetic diagnostics in fetuses with increased nuchal translucency or other ultrasound anomalies. The higher diagnostic yield and the reduced turnaround time attributable to this method, outweigh its potential to discover VOUS, which can be minimised by the concurrent parental analysis. Obtaining specific genetic diagnosis adds essential information about the potential full phenotypic expression beyond detected ultrasound anomalies and the postnatal prognosis. Furthemore, it has important information for future pregnancies and risk of recurrence in the families.

Additional diagnostic yield of array CGH in this reported series was 4,6%. There were three published studies of the diagnostic utility of microarrays in fetuses with ultrasound anomalies with a higher number of cases (Table 3) and they report diagnostic yield of 6,3% [5], and 6% [6, 7]. A slightly lower number in our report might be due to the inclusion criteria and categorization of cases. For example, in the study reported by Shaffer et al.[5] all pathogenic CNVs were considered in the reported percentages, including those bigger that 10 Mb. The diagnostic yield in our group of fetuses with multiple congenital anomalies was even higher (10,0%), which was expected, and reported by authors in the previous studies (9,5% [5], and 15,4% [13]).

**Table 3:** Diagnostic yield of aCGH in the prenatal diagnostics of the fetuses with ultrasound anomalies and/or increased nuchal translucency. In order to present the more relevant diagnostic yield evaluation, we only included the studies, reporting on more than 100 cases

First author	Number of samples with US anomalies or increased NT	Diagnostic yield (%)
Schaffer et al., 2012 [5]	2081	6.3%
Srebniak et al., 2016 [6]	957	6.0%
Wapner et al., 2012 [7]	755	6.0%
OUR STUDY	200	7,0%
Lee et al., 2012 [13]	180	11.1%
Yatsenko et al., 2013 [24]	162	5.0%
Armengol et al., 2012 [10]	159	5.7%
Rooryck et al., 2013 [25]	142	11.3%
Tyreman et al., 2009 [26]	106	6.7%
Ganesamoorthy et al, 2013 [27]	101	6,9%

The highest proportion of pathogenic CNVs was identified among the cases with musculoskeletal abnormalities. Allthough the numbers in our group were too small to make any further conclusions, the same finding was reported in the biggest study so far [5]. Among these cases we encountered two fetuses with thrombocytopenia-absent radius syndrome - TAR (OMIM#274000) and in both cases we confirmed compound heterozygosity for typical 1q21.1 deletion and the hypomorphic nucleotide change in the *RBM8A* gene on the other allele. Furthermore, we discovered an intragenic *NIPBL* deletion (Cornelia de Lange syndrome, OMIM#122470) in a fetus with reduction anomaly of the upper limbs and dysmorphic features. In the group of fetuses with the isolated increased nuchal translucency a small interstitial 16q24.1 deletion was identified, involving *FOXF1* gene (Alveolar capillary dysplasia with misalignment of pulmonary veins, ACDMPV, OMIM#265380) and *FOXC2* gene (Lymphedema-distichiasis syndrome, OMIM#153400). This is the first report linking the deleted *FOXF1* region to abnormal ultrasound findings in the first trimester. The post-mortem histopathology examination confirmed ACDMPV in the fetus. There has been one prenatal case reported in the literature with septated cystic hygroma, fetal hydrops, and a single umbilical artery presented at the 18th weeks of gestation, in which aCGH identified a deletion of 1,1 Mb in size, including both *FOXF1* and *FOXC2* [14]. In addition, postnatal ACDMPV with or without additional congenital anomalies has been shown to be linked to this region in 2009 [15]. Until recently the prenatal aCGH was predominantly used in the fetuses with structural ultrasound anomalies and only a minority of the studies reported results in the group of fetuses with the isolated increased NT. We expect to see other reports of the *FOXF1* deletions linked to increased NT.

Besides array CGH being the first tier test in the prenatal cases with abnormal ultrasound findings, it also proves to be crucial as a complementary method to classical karyotyping in specific prenatal scenarios. In our small cohort of 4 prenatal cases of seemingly balanced chromosomal rearrangements the unbalanced rearrangement was found in two cases. Furthermore, the identified rearrangements were both outside the putative breakpoints locations. Cryptic chromosomal rearrangements have been previously discovered not only at the translocation breakpoints, but elsewhere in the genome in the cases of apparently balanced karyotype, as well [16, 17]. This is highly relevant information in the group of fetuses with normal ultrasound results, where apparently balanced translocation is discovered after prenatal testing for other indications (maternal age or positive 1^st^ trimester screening results).

Although the aCGH is superior to the classical karyotype in most cases, the latter has proven to be essential to elucidate the mechanism, origin and recurrence risk in certain cases, as illustrated by the cases 5 and 22 (detailed in Table 2). These cases illustrate the importance of the fetal backup culture in order to have it available for the follow up studies, and highlights the need for integration of aCGH and cytogenetics.

Performing aCGH in prenatal settings introduces important challenges when genome wide approach is used, because of the potential to identify the CNV categorized as risk loci or VOUS. It is generally recommended, that VOUS and some of the low penetrance risk loci are not reported back to future parents. Some laboratories have their own national or institutional rules with respect to the reporting of these findings [9]. It is important to stress that when deciding not to report specific CNV, there should be different protocols implemented for the laboratory procedures, interpretation and reporting. Moreover, one should specify if such findings should be archived in the laboratory or directly to medical files. And if so, when to be communicated with the parents/patients. Due to these challenges and due to the fact, that parental samples are not collected concurrently, we reported back discovered VOUS. In our group of fetuses with ultrasound anomalies, altogether five CNVs classified as VOUS were discovered. After testing the parents it added additional information to these situations and indeed, in four out of five cases these were inherited from apparently healthy parent. In the fifth case (Case 25, Table 2) the pregnancy was terminated due to the type and severity of ultrasound anomalies.

Microarrays were introduced into the routine prenatal setting approximately 3–4 years ago [3–5], and at first used only in the case of fetuses with ultrasound anomalies or abnormal karyotype results. The method was proven to provide important additional diagnostic yield and some diagnostic centres also evaluated the method for testing the fetuses with increased nuchal translucency. Some countries went even further and implemented the aCGH for all invasive prenatal genetic testing [9]. Two different approaches are being discussed currently – one supports the implementation of the aCGH for all prenatal testing, regardless of the indication for genetic testing [18, 19]. Grande et al. [8] have shown that the clinically relevant CNV was present in 1,7% of pregnancies which underwent genetic testing due to advanced maternal age or positive aneuploidy screening (increased NT, abnormal result of maternal serum screening), thus justifying the use of the method in all invasive testing. The other perspective emphasizes the importance of and challenges introduced by the discovery of VOUS and so called risk factors – CNVs with non-fully penetrant and mild phenotype, usually inherited from a parent with minimal or no clinical features. It is suggested that these CNVs exert a phenotypic effect only in the presence of other genetic variants [20]. Both types of CNVs, the VOUS and risk factors, represent difficult counseling situations and place enormous pressure on the expectant parents. But the pretest counseling should include above mentioned findings, discuss that with the parents before the test and therefore minimize the chance of any undesirable situations. Finally, genome-wide aCGH may uncover findings of clinical significance, unrelated to the discovered ultrasound abnormalities, so called secondary findings. In our series of the prenatal cases we identified 6 such cases, all being a known non-fully penetrant microdeletion/microduplication syndrome.

## Conclusions

To conclude, the aCGH incrementally improved the diagnostic yield in routine prenatal genetic testing and proved to be an important first tier diagnostic test in the group of fetuses with ultrasound anomalies and the additional test in the fetuses with apparently balanced de novo chromosomal rearrangements. The pretest counseling sessions are essential and the potential discovery of VOUS and numerical aberrations with reduced penetrance should be discussed in advance with the future parents.

## Abbreviations

aCGH: Array comparative genomic hybridization
CNV: Copy number variant
DECIPHER: Database of chromosomal imbalance and phenotype in humans using Ensembl resources
IUGR: In utero growth restriction
NT: Nuchal translucency
TAR: Thrombocytopenia-absent radius syndrome
UPD: Uniparental disomy
VOUS: Variant of unknown significance
